# Type1 and 3 fimbriae phenotype and genotype as suitable markers for uropathogenic bacterial pathogenesis via attachment, cell surface hydrophobicity, and biofilm formation in catheter-associated urinary tract infections (CAUTIs)

**DOI:** 10.22038/IJBMS.2021.53691.12079

**Published:** 2021-08

**Authors:** Fatemeh Mohammad Zadeh, Hamed Zarei, Sahar Honarmand Jahromy

**Affiliations:** 1 Department of Microbiology, Varamin-Pishva Branch, Islamic Azad University, Varamin, Iran; 2 Department of Biology, Faculty of Basic science, Central Tehran Branch, Islamic Azad University, Tehran, Iran

**Keywords:** Biofilm, Catheterization, Fimbriae, Urinary tract infections, Uropathogenic

## Abstract

**Objective(s):**

Catheters are one of the factors for complicated urinary tract infections. Uropathogenic bacteria can attach to the catheter via cell surface hydrophobicity (CSH), form biofilms, and remain in urinary tract. The study was evaluated phenotypic and genotypic characteristics of fimbriae in *Klebsiella pneumoniae* and uropathogenic *Escherichia coli *(UPEC) isolates from patients with catheter-associated urinary tract infections (CAUTIs) and their association with biofilm formation.

**Materials and Methods:**

Urine specimens were collected through catheters in patients with CAUTIs. Sixty bacterial isolates were identified by biochemical tests. For determination of biofilm formation a tissue culture plate was used. Microbial adhesion to hydrocarbons (MATH) was conducted for CSH determination. The mannose-sensitive haemagglutination (MSHA) and mannose-resistant haemagglutination (MRHA) were determined for type 1 and type 3 fimbriae. Finally, the presence of genes encoding fimbriae was determined by PCR.

**Results:**

All isolates showed strong CSH, biofilm capacity and MRHA phenotype. The results showed that 20% of UPEC and 23% of *K. pneumoniae* isolates contained MSHA phenotypes. There was a significant association between biofilm formation and MSHA phenotype in UPEC isolates. The frequency of *fimA* (80%) and *fimH* (96.6%) in *K. pneumoniae* isolates was higher than UPEC isolates. Both types of bacterial isolates with MSHA phenotypes harbored the *fimH* gene.

**Conclusion:**

The phenotypic and genotypic characteristics of two bacterial species were highly similar. Also, the type of fimbriae affected bacterial biofilm formation through catheterization. It seems that *fimH* and *mrk* gene cluster subunits are suitable markers for identifying bacterial pathogenesis.

## Introduction

Urinary tract infections (UTIs) are the most common bacterial infections, affecting the bladder, kidneys, and urinary tract (1). They are often complicated by recurrent and chronic episodes (2). Studies have shown that up to 40% of all hospital-acquired infections such as nosocomial UTIs worldwide are commonly associated with catheterization (3), particularly catheter-associated UTIs (CAUTIs) (4). CAUTIs can lead to various complications, such as catheter encrustation, bladder stones, septicemia, endotoxic shock, and pyelonephritis (5). 

The most common uropathogenic Gram-negative bacteria in CAUTI cases include *Pseudomonas aeruginosa, Klebsiella pneumoniae, Proteus mirabilis, Proteus vulgaris, Escherichia coli, Citrobacter freundii, *and* Providencia rettgeri* (6). Although catheter is generally a critical indwelling medical device, its prolonged use for hospitalized patients allows bacteria to enter the bladder and form bioﬁlms through migration along the catheter surface (7). In urology, biofilm production can become a serious problem, and bacterial biofilms play an important role in CAUTIs (8). 

Biofilms include microbiologically derived sessile communities, characterized by cells that are irreversibly attached to the substratum, interface, or each other and are embedded in a matrix of extracellular polymeric substances (EPSs) (9). They are managed by physicochemical properties such as some interactions, namely, cell surface hydrophobicity (CSH). The hydrophobic properties of bacterial surfaces contribute to adhesion and binding to biotic and non-biotic surfaces. So, CSH plays an important role in bacterial colonization on different materials (10). 

Uropathogenic *E. coli *(UPEC) and *K. pneumoniae* as an important causes of CAUTIs have many adherence molecules such as pili, fimbriae, lipopolysaccharides, and capsular polysaccharides which manage bacterial adherence under different environmental conditions and organize biofilms (11, 12). Adhesion to the surface host urothelial via type 1 ﬁmbriae is the first step in UPEC bladder infection (13). *K. pneumoniae* have various chaperone-usher pili, contain type 1 and 3 pili (14). Type 1 pili have been characterized by mannose-sensitive hemagglutination (MSHA) phenotype but type 3 pili defined by mannose-resistant hemagglutination (MRHA) phenotypes with tannin-treated red blood cells (RBCs) (15). 

Studies have shown that type 1 pili of *K. pneumoniae* are highly homologous to those of UPEC. Type 1 pili are assembled via the chaperone-usher pathway (16). They are adhesive hair-like fibers, consisting of cylindrical pilus rods with fimA pilin subunits and small-tip fibrillae with fimF, fimG, and fimH components (17). *In vitro *studies showed that type 3 fimbriae that are encoded by the *mrk* operon cause attach to endothelial and bladder epithelial cell then lead to biofilm formation on abiotic surfaces and biotic surfaces (18). 

Type 3 ﬁmbriae at ﬁrst were identiﬁed in *Klebsiella, *but type 3 ﬁmbriae are commonly found in other members of the Enterobacteriaceae family. The *mrk* gene cluster (*mrkABCDF*) may be chromosome- or plasmid-borne (19). In some studies, reported that type 1 and type 3 ﬁmbriae have a role in enhancement of *K. pneumoniae* and subsequently bioﬁlm formation on urinary catheters in a catheterized bladder model (19). Therefore, the purpose of the present study was to explain the characteristics of *K. pneumoniae *and UPEC CSH, biofilm formation, and phenotyping and genotyping of their fimbriae to identify virulence factors associated with CAUTIs.

## Materials and Methods

***Patients and samples***

This descriptive cross-sectional study was conducted on 234 urine specimens, randomly collected from patients, who were referred to the Clinical Laboratory of Milad Hospital, Tehran, Iran, between September 2017 and June 2018. The inclusion criteria for the study was hospitalization in the units of the hospital. The urine samples were collected through the catheter port, using the aseptic technique or puncturing the catheter tube with a needle and syringe in patients with short-term catheterization (20). Catheter-associated asymptomatic bacteriuria (CA-ASB) is generally diagnosed when one or more organisms are present at quantitative counts ≥10^4^ CFU/ml in an appropriately collected urine specimen from a patient with no symptoms (21).

***Bacterial isolation and identification***

The specimens were inoculated on MacConkey agar and Eosin methylene blue( EMB) agar plates for detection of UPEC and* K. pneumoniae* isolates. Biochemical identification after overnight incubation at 37°C, was performed by culturing on triple sugar iron agar, sulfide indole motility medium, and Simmons’ citrate agar and conducting methyl-red/Voges-Proskauer (MR-VP) tests. All bacteriological culture media used in this study were purchased from Difco (Becton, Dickinson and Company, Franklin Lakes, NJ, USA). All isolates were suspended in 15% glycerol-supplemented Luria-Bertani (LB) medium and preserved frozen at −80°C.

***In vitro biofilm assay***

The tissue culture plate (TCP) method in tryptic soy broth (TSB) on a round-bottom 96-well microtiter plate (SPL Life Sciences, Korea) was used for determination of UPEC isolates biofilm formation (22). At first an overnight bacterial culture was grown in TSB (Merck, Germany) at 37°C, was equivalent to 0.5 McFarland standards (1.5x 10^8 ^CFU/ml) and diluted to 1:100 in TSB with 2% (w/v) glucose. 200 μl of cell suspension was transferred to a bottom well and were incubated at 37°C aerobically for 24 hr. Next, for removing non-adherent cells the culture was discarded, the wells of the plate washed three times with 200 μl of phosphate-buffered saline (PBS; pH=7.4; Sigma, USA), dried in an inverted position. For fixing and staining of adherent biofilm, it was fixed with 95% ethanol and stained with 0.1% crystal violet (Merck, Germany) for five minutes. 

After removing the unbound crystal violet stain, the wells were washed three times with sterile distilled water, and the microtiter plate was air-dried. The stained biofilm was suspended in 200 μl of glacial acetic acid (31). The optical density (OD) of each well was measured at 570 nm, using an ELISA plate reader (Cytation3, BioTek, USA). For biofilm formation assay the cut-off OD for a tissue culture plate is determined as three standard deviations above the mean OD of the negative control. Finally the biofilm production was deterd, according to the Stepanovic *et al.* study (23):

Optical density cut-off (ODc)= Average OD of negative control + 3× Standard deviation (SD) of negative control.

Each strain was tested in triplicate, and the wells with sterile TSB alone served as the controls. Each assay was performed in triplicate, and the mean values of crystal violet absorbance (±SD) were calculated for all replicates of the experiments.

***Bacterial CSH assay***

The Modified Microbial adhesion to Hydrocarbons (MATH) was used for assaying the hydrophobicity of bacterial cell suspensions as previously described by Rosenberg and Nwanyanwu *et al*. (24, 25). For the first stage after 24 hr of incubation of microbial cells, they were concentrated and harvested during the exponential growth phase followed by centrifugation at 5000× rpm for 20 min (TGL-16M, PR China). The PBS (7.6 g of NaCl, 1.9 g of Na_2_HPO_4_.7H_2_O, and 0.7 g of NaH_2_PO_4_.2H_2_O per liter; pH=7.2) was used as a hydrophilic solution. The suspensions were washed twice with PBS and the absorbance was measured at 660 nm as A1. For the second stage, 5 ml of microbial suspension and 1 ml of n-octane were mixed for 120 sec by vortexing. For ensuring that both solutions were separated in the biphasic state the mixture was incubated for 1 hr without shaking. By recording the changes in the absorbance of microbial suspensions due to microbial adhesion to n-octane at 660 nm, using a spectrophotometer (BioTek, USA) , the absorbance of the lower hydrophilic (aqueous) layer was calculated again as A2. Microbial CSH was expressed as adherence percentage (%Adh) and calculated using the following formula (26):

Adherence percentage= [(A1 - A2)/A1] × 100

The percentage of Adh at range of >50%, 20%-50%, and <20% classified the degree of hydrophobicity as strongly hydrophobic, moderately hydrophobic, and hydrophilic respectively (26).

***Phenotype determination of type 1 fimbriae by haemagglutination assay***

The hemagglutination assay was performed on a glass slide. Bacterial suspensions were prepared by growth in LB broth, LB agar, and TSA at 37 °C and mixed with an equal volume of 2% (v/v) guinea pig and rat erythrocyte suspensions in PBS (pH=7.4) in the presence or absence of 1% d-mannose. They were observed for hemagglutination over one minute. Hemagglutination was identified as mannose-resistant hemagglutination (MRHA) or mannose-sensitive haemagglutination (MSHA) in the presence or absence of mannose, respectively (27). The wells containing only the erythrocyte suspension, with or without D-mannose, served as the negative control, and *E. coli* ATCC 25922 was used as the positive control for MRHA.

***Phenotype determination of type 3 fimbriae by haemagglutination assay***

The type 3 fimbriae was detected by standard techniques, using erythrocytes treated with tannic acid (24). Human erythrocyte suspensions (3% v/v in saline) were prepared, and tannic-acid treatment of oxen erythrocytes (tanned oxen red cells), with or without 4% D-mannose, was performed. They were then mixed for 15 minutes in depressions of a tile at ambient temperature. Next, the erythrocytes were tanned by incubating equal volumes of 0.01% (wt/vol) tannic acid solution in saline and 3% erythrocyte suspension in PBS. The erythrocytes were subsequently washed twice in PBS (28). Type 3 pili displayed MRHA with tannin-treated RBCs.

***Molecular identification of mrkA, mrkB, mrkD, fimA, and fimH genes***

For molecular detection, before DNA extraction of all isolates by genomic DNA isolation kit (Gene Transfer Pioneers, Iran), Bacterial strains were cultured in LB at 37°C for 18 hr. The nucleotide sequences of primers that were used in this study are listed in Table 1 (29). The PCR material including 2.5 μl of 10X PCR buffer (Sinaclon, Iran), 2 mM MgCl_2_, 1 μm of each dNTP (Sinaclon, Iran), 2 U of Taq DNA polymerase (Sinaclon, Iran), 1 pmol of each primer, and 10 ng of bacterial DNA. So polymerase chain reaction (PCR) was conducted in a final volume of 25 μl. The amplification program was one min at 95°C, annealing for 55 sec at 58°C and for one min at 72°C, and a final extension at 72°C for five min. The amplification performed in 30 cycles. The PCR products were electrophoresed by gel agarose (Sinaclon, Iran) and visualized by a UV transilluminator (UVT-20M, Kigen).

***Statistical analysis***

Data were collected and entered in a database using Microsoft Excel 2013. Statistical analysis was performed in SPSS version 20 (SPSS, Inc., Chicago, IL, USA). Chi-square test was used for comparison of categorical variables. The relationships between variables and 95% confidence intervals were also calculated. *P*-value less than 0.05 was considered statistically significant. 

## Results

Of 234 urine samples collected from patients under study, 110 (47%) patients with CAUTIs (68 female with CAUTI and 42 male with CAUTI) were detected. Based on the results of biochemical tests a total of 30 UPEC and 30 *K. pneumonia *strains were detected and selected randomly. 

***Biofilm formation***

Based on the standard microtiter plate method and the OD cut-off value for UPEC and isolates (Table 2), there were 15 (50%) strong, 7 (23.3%) moderate, and 8 (26.7%) weak biofilm producers among UPEC isolates; also, non-biofilm-producing bacteria were observed. On the other hand, there were 18 (60%) strong, 8 (26.70%) moderate, and 4 (13.30%) weak biofilm producers among *K. pneumoniae *isolates. 

***Hydrophobicity determination***

The degree of cell hydrophobicity on the MATH assay, as shown in Table 3, revealed that all UPEC and* K. pneumoniae *isolates had strong CSH. The results showed that all isolates with a biofilm formation capacity had strong CSH.

***Type 1 fimbria determination***

**Figure 1:**
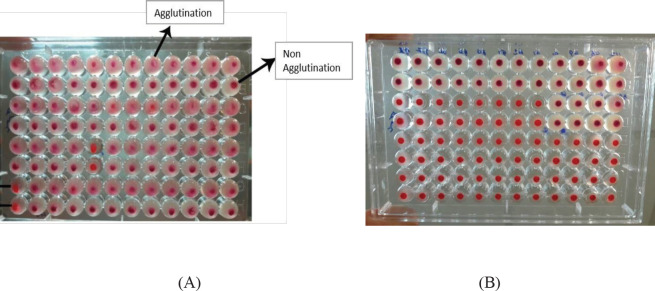
Haemagglutination test: A) Type 1 fimbria (MSHA) determination; and B) type 3 fimbria determination (MRHA)

One of the indirect indicator for the presence of fimbriae among UPEC isolates is the ability of bacteria to cause agglutination of erythrocytes. Since type 1 fimbriae are sensitive to mannose sugar, if agglutination is formed in the absence of mannose sugar, but not in the presence of mannose sugar, it is confirmed that there are type 1 fimbriae (Figure 1A). Among UPEC and* K. pneumoniae* isolates, 6 (20%) and 7 (23.3%) isolates showed type 1 fimbriae phenotypes (MSHA), respectively. 

***Type 3 fimbria determination***

Since type 3 fimbriae are resistant to mannose sugar, if agglutination is formed in the absence and presence of mannose sugar, the presence of type 3 fimbriae is confirmed (Figure 1B). All UPEC and* K. pneumoniae* isolates showed type 3 fimbria phenotypes (MRHA). 

Overall, 6 (20%) and 7 (23.3%) UPEC and* K. pneumoniae* isolates showed both MSHA and MRHA phenotypes, respectively. 

***Relationship between the phenotypic type of fimbriae and biofilm formation capacity in UPEC and K. pneumoniae isolates***

The frequency of type 3 fimbriae in both isolates was 100%, based on the biofilm formation criteria. According to these criteria, among UPEC isolates, the frequency of type 1 fimbriae was 33.3% and 12% in strong and moderate biofilm producers, respectively. There were no type 1 fimbriae among weak biofilm producers. Data analysis showed a significant association between biofilm formation and MSHA phenotypes in UPEC isolates (*P=*0.0031) (Table 4). Based on the biofilm formation criteria, among *K. pneumoniae* isolates, the frequency of type 1 fimbriae was 6.6%, 42.8%, and 25% for strong, moderate, and weak biofilm producers, respectively (Table 4). However, there was no significant relationship between biofilm formation and MSHA phenotype in* K. pneumoniae* isolates (*P*≥0.05).

***Molecular identification of mrkA, mrkB, mrkD, fimA, and fimH genes***

**Figure 2:**
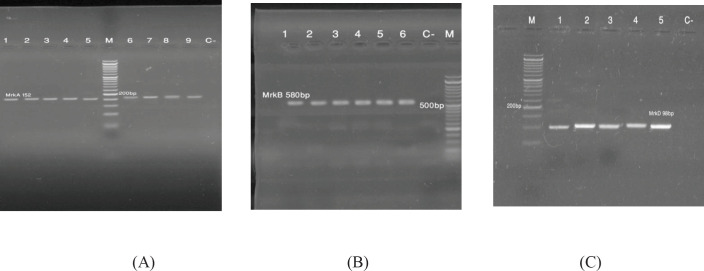
Gel electrophoresis of PCR products: (A) Lanes 1-9: mrkA gene; (B) Lanes 1-6: mrkB gene; and (C) Lanes 1-5: mrkD gene (C-: negative control; M: 50 bp ladder)

The results of analysis of PCR amplification products by gel electrophoresis showed different band sizes for genes encoding type 3 fimbriae: *mrkA* gene, 152 bp (A); *mrkB* gene, 580 bp (B); and *mrkD* gene, 98 bp (C) (Figure 2). 

Figure 2: Gel electrophoresis of PCR products: (A) Lanes 1-9: *mrkA* gene; (B) Lanes 1-6: *mrkB* gene; and (C) Lanes 1-5: *mrkD* gene (C^-^: negative control; M: 50 bp ladder).

**Figure 3:**
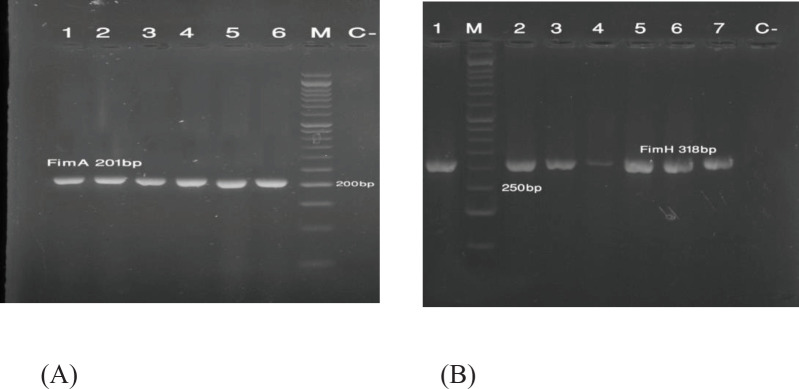
Gel electrophoresis of PCR products: (A) Lanes 1-6: fimA gene; and (B) lanes 1-7: fimH gene (C-: negative control; M: 50 bp ladder)

The analysis of PCR amplification products for genes encoding fimbriae type 1 via gel electrophoresis showed bands with different sizes: *fimA *gene, 201 bp (A); and *fimH* gene, 250 bp (B) (Figure 3). 

**Figure 4:**
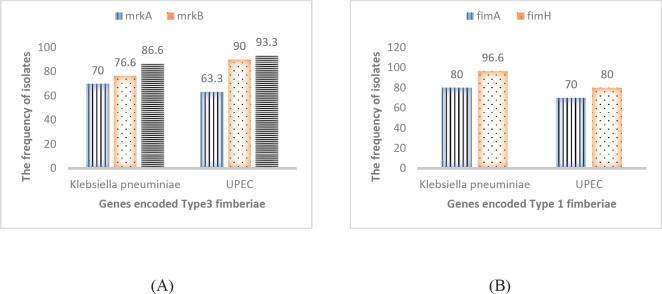
The frequency of genes encoding type 3 fimbriae (A) and type 1 fimbriae (B) in *Klebsiella pneumoniae *and *Escherichia coli *(UPEC) isolates

As shown in Figure 4A, the frequency of *mrkB* and *mrkD* genes was higher in UPEC isolates (90% and 93.3%, respectively) than *K. pneumoniae* isolates (76.6% and 86.6%). There was no significant difference in the frequency of these genes between both bacterial species (*P*≥0.05). Also, the frequency of *fimA *(80%) and *fimH* (96.6%) genes among *K. pneumoniae* isolates was higher than UPEC isolates (70% and 80%, respectively) (Figure 4B).

The results showed that among *K. pneumoniae *isolates, the frequency of gene clusters, such as *fimA*+*fimH* (76%), was higher than similar clusters in UPEC isolates. However, among UPEC isolates, the frequency of *mrkB*+*mrkD* gene cluster (86.6%) was higher than others. The frequency of isolates harboring genes, encoding type 1 and type 3 fimbriae simultaneously, was higher among *K. pneumoniae *isolates (36.6%), compared to UPEC isolates (20%). The frequency of *mrkA*+*mrkB*+*mrkD* gene cluster was relatively similar in the two bacterial isolates (50% and 53.3% in *K. pneumoniae *and UPEC isolates*, *respectively).

As shown in Table 5, the frequency of *fimA *(80%) and *fimH *(86.6%) genes was high in UPEC isolates with a strong biofilm formation capacity (Table 5). Data analysis showed a significant association between *fimA* gene and biofilm formation (*P*=0.0011) in UPEC isolates. Also, among UPEC isolates, the frequency of *fimA* gene was lower in weak biofilm producers. All isolates with MSHA phenotypes harbored *fimH* gene (Table 5). 

Among *K. pneumoniae* isolates, the frequency of *fimA* and *fimH* genes was relatively similar in all groups of biofilm producers (Table 5). All *K. pneumoniae* isolates harbored *fimH* genes. The frequency of *mrkA* (0%) and *mrkD* (50%) genes was lower among weak biofilm producers than other biofilm producers. The frequency of *mrk*+*fim* gene cluster was 50%, which is higher than moderate biofilm producers. However, there was no gene cluster in weak biofilm producers.

## Discussion

In this study, the prevalence of CAUTIs among patients hospitalized in Milad Hospital, Tehran, Iran, was 47%. Complicated UTIs are defined as infections that are related to the urinary tract and are associated with urinary obstruction, retention due to neurological diseases, immunosuppression, kidney failure, kidney transplant, and the presence of foreign body, like catheters (30). Catheters are one of the predisposing factors for complicated UTIs that may lead to the release of blood and sepsis. Uropathogenic bacteria can attach to the catheter and form biofilms (31). 

Previous studies have shown that more than 50% of patients are catheterized during hospitalization, which severely increases the risk of microorganism colonization in hospitalized patients. UPEC and *K. pneumoniae *are common causes of CAUTIs (32). In the present study, all *K.* *pneumoniae* and UPEC strains that were isolated from CAUTIs were biofilm producers. The prevalence of strong biofilm producers among both bacterial species was higher than the other two biofilm-producing groups (50% and 60%, respectively). *In* *vitro* studies have demonstrated a biofilm formation rate of 40% in *K. pneumoniae* isolates from clinical specimens such as urine, sputum, blood, and wound swabs. Also, Niveditha *et al*. in 2012 showed that 63% of *K. pneumoniae* isolates from the urine specimens of catheterized patients with UTIs were positive for *in vitro* biofilm formation (31). 

In a study by Shah *et al*. in 2019 on UPEC isolates, biofilm formation was confirmed in the majority of UPEC (62%) isolates (32), which is in agreement with other studies by Karam *et al*. in 2018 (33). Also, a study by Fattahi *et al*. indicated a higher rate of biofilm formation (90%), which is in agreement with our results (34). Bacterial attachment is the first and the most important step in biofilm formation. CSH plays a critical role in the establishment and attachment of bacteria to living and non-living surfaces, such as catheters, implants, and artificial heart valves that are made of hydrophobic materials, causing hydrophobic microorganisms to bind to these surfaces easily (35). 

In the present study, all *K. pneumoniae* and UPEC isolates showed strong CSH, based on the MATH assay. Also, Gogra *et al*. in 2010, using the MATH assay, compared the prevalence of *Escherichia coli* with 96.9% cell surface hydrophobicity to 78.2% for *Staphylococcus aureus* and 50.30% for *Aspergillus* species (36). In 2018, a meta-analysis by Mirani *et al*. showed that CSH and bacterial growth were involved in the formation of *Pseudomonas aeruginosa, S. aureus*, and *E. coli* biofilms (37). Moreover, according to *in vitro* studies of various bioﬁlm models, the attachment of bacteria to catheters was initiated by adhesions. For example, ﬁmbriae, such as type 1 and type 3 fimbriae, were located on the bacterial surface. 

The UPEC and *K. pneumoniae* genomes encode various adhesive ﬁmbriae that facilitate attachment to the uroepithelial surface and enable bacteria to resist elimination through the urine ﬂow. In the present study, 6 (20%) and 7 (23.3%) UPEC and* K. pneumoniae* isolates showed type 1 fimbrial) MSHA (phenotypes, whereas all bacterial isolates showed type 3 fimbrial (MRHA) phenotypes. It has been shown that the expression of type 1 pili is strictly controlled by phase variation, which reversibly switches between active type 1 pilus expression (Phase-ON, piliated cells) and loss of expression (Phase-OFF, non-piliated cells) (38). 

Burmolle *et al*. in 2008 demonstrated that type 3 fimbriae have intermediate adherence to the endothelial and epithelial lines of the bladder and form biofilms on surfaces (39). Among UPEC isolates, based on the biofilm formation criteria, the frequency of type 1 fimbriae was 33.3% and 12% in strong and moderate biofilm producers, respectively. However, there were no type 1 fimbriae in weak biofilm producers. There was a significant association between biofilm formation and MSHA phenotypes among UPEC isolates. Also, among *K. pneumoniae* isolates, based on the biofilm formation criteria, the frequency of type 1 fimbriae was 6.6%, 42.8%, and 25% for strong, moderate, and weak biofilm producers, respectively. There was not association between biofilm formation and MSHA phenotype in* K. pneumoniae* isolates. 

In some studies *E. coli*, as a closely related species to *K. pneumoniae*, type 1 fimbriae were shown to promote biofilm formation. This significant difference may be related to the characteristic production of high amounts of capsular materials by *K. pneumoniea, *as capsule expression inhibits type 1 fimbriae functionally (40). In this regard, Shah *et al*. in 2019 reported the expression of MRHA and MSHA in *E. coli* uroisolates in 52.3% (n=55) and 5.7% (n=6) of isolates, respectively (32). Also, a study by UIeIIt *et al*. in 2007 showed that 95% of *K. pneumoniae* isolates expressed type 3 fimbriae, which is in agreement with the current study (41). In this study, it was clear that both bacterial species with strong CSH contained MRHA phenotypes. 

Type 3 fimbriae enhance the adhesion of *K. pneumoniae* to non-biological surfaces by increasing hydrophobicity. This characteristic can be attributed to the high percentage of hydrophobic amino acids in the main unit of *mrkA* gene (42). In 2016, a study by Stæk *et al*. demonstrated that type 1 fimbriae in UPEC isolates contributed to the ability of bacteria to bind to and invade human bladder cells (43). In this regard, Schroll in 2010, by using isogenic mutants, found that type 3 fimbriae, but not type 1 fimbriae, strongly promote biofilm formation in *K. pneumoniae* isolates (17). 

Conversely, Caitlin *et al*. in 2013, by performing mutation experiments, found that both ﬁmbrial types enhanced colonization and persistence of CAUTIs due to *K. pneumniae *(45)*. *The frequency of *fimA *(80%) and *fimH* (96.6%) genes in *K. pneumoniae* isolates was higher than that of UPEC isolates (70% and 80%, respectively). Among UPEC isolates, the frequency of *mrkB* (90%) and *mrkD* (93.3%) genes was higher than *K. pneumoniae* isolates (76.6% and 86.6%, respectively). There was no significant difference in the frequency of genes between bacterial isolates. The results showed that among *K. pneumoniae *isolates, the frequency of gene clusters, such as *fimA*+*fimH* (76%), was higher than similar gene combinations in UPEC isolates. 

The frequency of *mrkA*+*mrkB*+*mrkD* gene cluster was relatively similar in both bacterial isolates (50% and 53.3% in *K. pneumoniae *and* UPEC *isolates*, *respectively). Also, among UPEC isolates, the frequency of *fimA* genes was lower in weak biofilm producers. All UPEC and *K. pneumoniae *isolates with MSHA phenotypes harbored *fimH* gene. It was found that the MSHA phenotypes of UPEC and *K. pneumoniae *isolates, expressing type 1 pili, require *fimH* adhesion. In this regard, Mahmood *et al*. in 2015 revealed that all biofilm-producing *E. coli* (n=15, 100%) isolates from UTI samples were positive for *fimH* gene, whereas all biofilm-producing *K. pneumoniae* isolates from UTIs and diabetic foot infections (n=9, 100% and n=5, 100%, respectively) were positive for *mrkD* gene. 

**Table 1:** TThe nucleotide sequences of *mrkA*, mrkB, *mrkD*, *fimA*, and *fimH* primers

**Band size**	**Nucleotide sequences**	**Primers**
580 bp	CACGGTTTTGCTGTTCAGGG	*mrkB-F*
	TAT CGG TGG AGA GAA CCA GC	*mrkB-R*
980 bp	AAC GTC ATG GGC ATC AT	*mrkD-F*
	TCC CTA CTG GTA AGT CCG GG	*mrkD-R*
152 bp	TGA TGG CAC CAA ACA GGA TGA	*mrkA-F*
	TAT CGG TGG AGA GAA CCA GC	*mrkA-R*
201 bp	CAT CCG CGT TCG CTA TAC CA	*fimA-F*
	TTC TGG CCC TGC AAA ACT CT	*fimA-R*
318 bp	GAT CAC CGA CTA CGT GAC CC	*fimH-F*
	GGG ACC ACC ACG TCG TTA TT	*fimH-R*

**Table 2:** Interpretation of biofilm production of UPEC and *Klebsiella pneumoniae* isolates

Average OD	**average OD of bacteria**		Biofilm production
	*UPEC*	*Klebsiella pneumonia*	
OD≤ODc	OD≤0.1629	OD≤0.1647	Non-adherent
ODc<OD≤ 2×ODc	0.1629<OD≤0.3258	0.1647<OD≤0.3294	Weakly adherent
2×ODc<OD≤ 4×ODc	0.3258<OD≤0.6516	0.3294<OD ≤0.6588	Moderately adherent
4×ODc<OD	0.6516<OD	0.6588<OD	Strongly adherent

*Uropathogenic *Escherichia col*i, Mean: 0.1325, SD: 0.01014, ODc: 0.1629.

*Klebsiella pneumonia, Mean: 0.1131, SD: 0.0172, ODc: 0.1647.

SD: Standard Deviation, ODc: Optical density cut-off

**Table 3.** The degree of cell surface hydrophobicity (CSH) in uropathogenic *Escherichia coli *(UPEC) and *Klebsiella pneumoniae*

**Assay **	**Criteria**	**Hydrophobicity**	**No. of bacterial isolates (%)** **UPEC ** ***Klebsiella pneumoniae***	
MATH	>50%	Strongly hydrophobic	30 (100)	30 (100)
	20-50%	Moderately hydrophobic	0	0
	<20%	Hydrophilic	0	0

UPEC: Uropathogenic* Escherichia coli*; MATH: Microbial adhesion to hydrocarbons

**Table 4:** Association between the phenotypic type of fimbriae and biofilm formation capacity in uropathogenic *Escherichia coli *(UPEC) and* Klebsiella pneumoniae* isolates

**Phenotypic type of fimbriae**		***Klebsiella pneumonia *** **(n=30)** **N (%)** **Biofilm criteria**		**UPEC (n=30)** **N (%)** **Biofilm criteria**		
	Strong producer (n=18)	Moderate producer (n=8)	Weak producer (n=4)	Strong producer (n=15)	Moderate producer (n=7)	Weak producer (n=8)
Type 1 fimbriae	6 (33.3)	1 (12)	0	1 (6.6)	3 (42.8)	2 (25)
Type 3 fimbriae	18 (100)	8 (100)	4 (100)	15 (100)	7 (100)	8 (100)

**Table 5:** The frequency of genes encoding fimbriae based on the biofilm formation criteria and fimbrial phenotypes in *Klebsiella pneumoniae* and uropathogenic *Escherichia coli *(UPEC) isolates

**Bacterial isolates**	**Genes** **encoding fimbriae**		**Biofilm criteria**		**Fimbriae type**	
		**Strong**	**Moderate**	**Weak**	**Type 1 fimbriae**	**Type 3 fimbriae**
	*fimA*	*12(80)	5(71.4)	3(37.5)	3(50)	-
**UPEC**	*fimH*	13(86.6)	5(71.4)	6(75)	6(100)	-
	*mrkA*	10(66.6)	4(57.1)	4(50)	-	19(63.3)
	*mrkB*	13(86.6)	6(85.7)	7(87.5)	-	17(56.6)
	*mrkD*	14(93.3)	6(85.7)	8(100)	-	28(93.3)
	*Fim+mrk*	5(3.3)	1(14.2)	0	-	-
***Klebsiella pneumonia***	*fimA*	15(83.3)	6(75)	3(75)	6(100)	_
	*fimH*	17(94.4)	8(100)	4(100)	7(100)	-
	*mrkA*	15(83.3)	6(75)	0	-	20(66.6)
	*mrkB*	14(77.7)	5(62.5)	4(100)	-	22(73.3)
	*mrkD*	17(94.4)	7(87.5)	2(50)	-	25(83.3)
	*Fim+mrk*	9(50)	2(25)	0	-	-

## Conclusion

We found that the prolonged use of urinary catheters, CSH, and subsequently, the biofilm-forming capacity of UPEC and *K. pneumoniae* isolates are important risk factors for developing CAUTIs. The common recurrence of CAUTIs causes antibiotic treatment failure, which leads to re-colonization of the urinary tract by organisms that survive in the catheter bioﬁlm. Therefore, rapid removal of catheters or development of catheter surfaces, which decrease the attachment of hydrophobic bacteria and for a better understanding of the catheter attachment mechanisms, the study of surfaces adhesion such as fimbriae is recommended. For this purpose, the role of type 1 fimbriae, with biofilm formation ability, was demonstrated in UPEC isolates, and it was found that all *UPEC* and *K. pneumoniae *isolates with MSHA phenotypes harbored *fimH* gene. On the other hand, both bacterial isolates contained type 3 fimbriae (MRHA phenotype) and harbored *mrk* genes with similar frequencies. The phenotypic and genotypic characteristics of these two bacterial species were very similar. This finding revealed that the *mrk* gene cluster originally belongs to the genetic structure of *K. pneumoniae *and is horizontally transferred to UPEC. In future studies, the evaluation of genotypic expression of these genes is recommended for a better understanding of uropathogenic bacteria behaviors, such as biofilm formation. One of the future approaches can be the use of fimbrial subunits as suitable markers to identify the pathogenesis of these bacteria and to propose a proper vaccine candidate to prevent UTIs.
